# Dental Ethics

**Published:** 1871-08

**Authors:** E. M. Morrison


### DENTAL ETHICS.

BY E. M. MORRISON.

**Read before the Indiana State Dental Association, at Fort Wayne, June 27th, 1871.**

According to Dunglison, Bentham introduced the word deontology, to signify morals, or the science of duties.

Medical deontology defines the duties and rights of medical practitioners. The word *ethics*, however, has become generally in use in the place of deontology, and will be adhered to in this paper. Then dental ethics is a system of rules to regulate the actions, rights, manners, and duties of dental practitioners.

The subject is of much importance to the profession, but I fear that it has been too little observed by many of us, yet it is essential that our code of ethics be uniform, and well lived up to. To secure these ends, as far as possible, the American Dental Association has adopted a code of ethics, very good in its main features, and I am glad that it has been accepted by nearly all reputable dental societies of the United States.

Do we live according to the discipline? Do we practice according to the code? Do not some dentists encourage hopes of success, when in the nature of the case there is uncertainty? Are we temperate in all things? Do all of us feel bound at all times to maintain the honor and dignity of the profession? Do not some speak in disrespect of their brethren? Do we not see public advertisements, cards, handbills, posters and signs, calling attention to peculiar styles of work, lowness of prices, special modes of operating, and claiming superiority over neighboring practitioners? Do we not see published reports of cases, or certificates in public prints, and men going from house to house to solicit or perform operations, circulate and recommend nostrums? Finally, have not many of us seen nearly every provision of the code violated, by some who are yet members of respectable societies, and not raised our voices in condemnation?

All this is wrong. We should not hesitate to call in question the acts of any member of the profession, who makes a "*departure*" from the code, or violates the rules which all should observe.

To purge our societies of all those who violate the code, and play the part of the quack or montebank, would be as a refiner's fire, to purify and strengthen us in the eyes of an appreciating public.

Then, we should elevate our standard of ethics. Article III of our code, commences as follows: "Dental Surgery is a specialty in medicine," and it goes on to define the relations of the dentist and the general practitioner.

Now, if we could only realize the fact, that we are physicians in good standing, practicing our specialty, we would be elevated as a part of that profession. Dentistry, however, has not been so looked upon by medical men generally, and to our disgrace, they often employ the traveling tooth carpenter in their families in place of the man of science and skill. Physicians who could not be induced to violate the code in relation to the general practitioner, do not see dentists as part of their profession, simply because our code does not come up to the proper standard, and all dentists are seen upon the same low ground. When we come out of the mire and the bog, the general practitioner will see us as his equal. Our relations will be mutual, and our services to each other will be as medical men to medical men. We will then have the influence of the medical profession generally in the suppression of dental quackery, rather than in its support as now. If we are a part of the medical profession, we must come up to the standard of that profession in ethics and learning. If not, we should not make such pretensions, but be content with a code commensurate with our low estate.

There should be an article added to the code of ethics, to check the disgraceful lust for large profits from the profession, through the medium of patents.

In the code of ethics of the American Medical Association, we find the following section:

"Equally derogatory to professional character is it for a physician to hold a patent for any surgical instrument or medicine, or to dispense a secret nostrum, whether it be the composition or exclusive property of himself or others. For if such nostrum be of real efficacy, any concealment regarding it, is inconsistent with beneficence and professional liberty; and if mystery alone gives it value and importance, such craft implies either disgraceful ignorance or fraudulent avarice. It is also reprehensible, for physicians to give certificates attesting the efficiency of patent medicines, or in any way to promote the use of them."

This section should be incorporated into our code of ethics, with such verbal changes as to make it applicable.

Dr. J. P H. Brown, (Dental Register, vol. XXIII, pp. 12 and 13,) makes the following remarks, which are so well to the point that I copy them here:

"In the first place, dentistry is unquestionably as much a part of medicine as surgery. It is so considered by our first physicians and surgeons, and so proclaimed by our dental professors. Dentistry being a part of medicine, it necessarily follows that the same rules and etiquette which are laid down to govern the professional conduct of practitioners of medicine and surgery, should also govern the practitioners of dental science.

"As the medical profession owes its power, influence and skill, to the perfectly free interchange of ideas and opinions, it is held to be derogatory for one of its members to patent any remedy, appliance, or principle of its application. As each member draws from the common fund of knowledge—the accumulated discoveries of centuries—he is morally and professionally bound to contribute his portion, either small or great, to the common stock."

The same is true of our specialty, and in this respect we have recently one of the noblest examples, that of Dr. Barnum, with his coffer dam, any where to be found. All honor is due him for his good example, and great contribution to our profession. He has had the nerve to do right, and his good example should ever be a rebuke to patent mongers, who wish to fill their coffers at the expense of co-workers, and true progress.

In the Dental Register, (vol. XXIII, p. 50,) in relation to a patented article, which the patentee wished to perfect before putting into the market, the Editor says: "We are not certain that this is the best course; if fifty or a hundred experimenters were at work, the progress most probably would be far more rapid; at least it has proved so with other things."

Why, then, have the progress of the profession checked for the benefit of one individual?

We can not afford to pay patentees for their patents to experiment with; neither can we experiment without paying for the privilege, so that a complete check is put upon the progress demanded by the exigencies of the times.

It is easy to see the drift of such a course. It will carry us down to the condition of mere mechanics. Even now, some have come down in their feelings, and look out from a mechanical stand point.

Dr. P. C. Branch, (*Dental Cosmos*, vol. XI, p. 76,) says: "Now I believe that a proper condition of things can only be restored by a change in the relations of these two branches of dentistry. Let us cease to regard mechanical dentistry as professional labor, and throw it open to competition like any other "trade," (if you will,) and then by employing mechanics at mechanics wages, underbid and drive the humbugs that are everywhere to be found, from the field, with the irresistible argument of "the cheapest and the best." Thus the mechanical department will come under the supervision of the professional; thus the professional in the exercise of good common sense, and enterprising business qualifications, can make the mechanical its willing servant, unable to exist without the oversight of its master."

It is difficult to see so clearly which would be the master, and which the obedient servant.

If mechanical dentistry is to come down to the level of a trade, I will abandon that part of my profession, for I hold that a profession can not be a mere trade in one of its departments, and make that department subservient to the other. A profession can not govern a trade any more than a trade can govern a profession. I can not contemplate such a separation, however, though we have been loaded down with patent after patent, and with quacks and charlatans, till they are legion. It is clear that the profession is progressive in its general bearing. While we feel the pressure of mechanical and patent degradation, and are mortified at the sight of flaming handbills and posters by the way side and in high places, we are at least keeping pace with the balance of mankind in the way of progress.

Dr. W. H. Trueman, (*Dental Cosmos*, vol. XI, p. 174,) says: "The advance in mechanical dentistry would no doubt have been more rapid than it has, if all the talent it has enlisted had been directed more to the discovery of new and the improvement of old ideas, and less to the rapid accumulation of wealth. Those practitioners who can take a retrospect of two decades placed side by side, can not have failed to observe how often its advance has been checked by those who have strength of body and mind to occupy the front ranks, desiring to be carried on the shoulders of their weaker brethren as a reward for some real or fancied advance they have made, nor yet can they fail to have noticed how often this ungenerous conduct has resulted in a fall so severe as to consign the unfortunate to a place among the limping and lame ever after."

Dr. Trueman was here speaking of the patent spring plate, and there can be no doubt of his allusion to patents and patentees.

Dr. John Allen has said the most foolish act of his life was in procuring a "patent for his continuous gum process," so I hope we will merge from the gorges, to which we have been dragged by avarice and patent mongers, and ascend to the hight demanded by our specialty.

Let there be thoroughly organized societies all over the country, working in unison, and every year will mark a progress so well established that no outside pressure can force a retrograde movement.

